# A single, extinction-based treatment with a kappa opioid receptor agonist elicits a long-term reduction in cocaine relapse

**DOI:** 10.1038/s41386-017-0006-4

**Published:** 2018-02-22

**Authors:** Jasper A. Heinsbroek, Amelia B. Furbish, Jamie Peters

**Affiliations:** Department of Neuroscience, Medical University of South Carolina Charleston, Charleston, SC 29425 USA

## Abstract

Kappa opioid receptor (KOR) agonists have known anti-addiction properties and can reduce drug seeking. Their potential for clinical use has largely been daunted by their aversive properties mediated through p38 MAPK signaling. Here we examined the therapeutic potential of the KOR agonist U50,488 (U50) to reduce cocaine seeking in a self-administration model. Following cocaine self-administration and 7 days of forced home-cage abstinence, rats were administered a single dose of U50 (5 mg/kg, i.p.) 30 min prior to the first extinction training session, wherein cocaine and the discrete cocaine-paired cues were no longer available. U50 reduced cocaine seeking on this first extinction session, but did not alter extinction training over subsequent days. 2 weeks after U50 treatment, rats underwent a test of cue-induced reinstatement, and rats that had received U50 reinstated less than controls. Central inhibition of p38 MAPK at the time of U50 administration prevented its long-term therapeutic effect on reinstatement, but not its acute reduction in drug seeking on extinction day 1. The long-term therapeutic effect of U50 required operant extinction during U50 exposure, extended to cocaine-primed reinstatement, and was not mimicked by another aversive drug, lithium chloride (LiCl). These data suggest U50 elicits its long-term anti-relapse effects through a KOR-p38 MAPK-specific aversive counterconditioning of the operant cocaine-seeking response. A single, albeit aversive treatment that is able to reduce relapse long-term warrants further consideration of the therapeutic potential of KOR agonists in the treatment of addiction.

## Introduction

Cocaine relapse can be triggered by multiple factors, including conditioned cues that act as reminders of the drug experience, and stress [1]. The kappa opioid receptor (KOR) system may influence drug seeking by virtue of its prominent role in stress [2]. However, the complexity of this system is underscored by reports that both KOR agonists and antagonists reduce drug seeking in models of cocaine relapse [3–5]. KOR agonists elicit effects similar to stress in rodents, such as dysphoria and/or aversion [6,7], depressive symptoms such as elevated reward threshold [8], and antinociception [9,10]. Indeed, the clinical use of KOR agonists in humans has been limited by these known aversive properties [11,12].

KOR activation can trigger various signaling cascades, including those mediated directly through G-proteins, and indirectly through the recruitment of beta arrestins. Beta-arrestin signaling activates p38 mitogen-activated protein kinases (MAPK), which has been specifically implicated in the aversive and dysphoric effects of KOR agonists [6,7,13–15]. Thus, current treatment strategies are focused on the development of functionally selective or biased agonists that avoid the beta-arrestin/p38 MAPK signaling pathway, to improve tolerability [16–19]. By extension of this same logic, p38 MAPK inhibitors co-administered with KOR agonists, a strategy we employed in the present study, should prevent KOR-mediated aversion, and indeed prevent U50-induced conditioned place aversion (CPA) [7].

Stress causes release of corticotropin-releasing factor, which in turn induces dynorphin release and subsequent KOR activation [19,20]. Consistent with the notion that KOR activation emulates a stressor, KOR agonists can induce reinstatement of drug seeking [21,22]. KOR antagonists reduce depressive symptoms and stress-induced cocaine seeking in preclinical models [3,23]. Despite these promising therapeutic advantages, KOR antagonists may not be effective in reducing other forms of relapse, such as cocaine-primed reinstatement [3].

By contrast, KOR agonists reduce both cocaine taking and cocaine-primed reinstatement acutely, during KOR agonist exposure [4,5,24–26]. Thus, KOR agonists may be particularly therapeutic when combined with cocaine, perhaps through their known ability to oppose cocaine reward [8]. Repeated KOR agonist exposure, on the other hand, can result in opposing effects on the dopamine system [27], and desensitization of the KOR [28], Few studies have examined the long-lasting effects on drug seeking after a single administration of KOR agonist [26], or effects of KOR agonists on extinction and cue-induced reinstatement. The present study examined the ability of a single acute dose of the KOR agonist U50,488 (U50) to enhance extinction and reduce cue-induced reinstatement of cocaine seeking in the long term.

## Materials and methods

### Subjects

Male Sprague-Dawley rats (72 total; Charles River Laboratories) weighing 250–275 g on arrival were individually housed in a temperature and humidity controlled environment with a 12 h light/dark cycle (6:00 a.m. lights off). Experiments were conducted during the rats’ dark cycle. Rats were food-restricted to 20–22 g of food per day (80–95% of free-feeding body weight) to promote behavioral performance. Water was available ad libitum in the home cage. Seven rats were eliminated from the final analysis due to misinjection of drug (*n* = 2), statistical outliers defined as more than 2 standard deviations beyond the mean (*n* = 3), and infusion problems (*n* = 2).

### Drugs

Cocaine hydrochloride (National Institute on Drug Abuse Drug Supply Program) was dissolved in 0.9% saline. U50,488 (U50) (racemic; Tocris) was dissolved in water and administered at a dose of 5 mg/kg (1 ml/kg, i.p.) based on previous studies [7,29]; notably, U50 has a half-life of 8 h [30]. SB203580 (Calbiochem) was dissolved in 3.8% dimethyl sulfoxide/saline to produce a 1 mM stock solution. The concentration and infusion volume were based on previous effective doses administered intracerebroventricularly (icv) in rodents [7,31]. Lithium chloride (LiCl; Sigma-Aldrich) was dissolved in sterile saline (0.6 M solution) and injected at 5 ml/kg based on previous studies [32,33].

### Surgery

Surgery was performed under ketamine/xylazine (100/6.7 mg/kg) anesthesia, and ketorolac (15 mg/kg) was administered for analgesia. Rats were implanted with intravenous catheters for subsequent cocaine self-administration as described previously [34], and an intracranial guide cannula (26 gauge) was stereotaxically implanted above the right lateral ventricle using the following coordinates (from bregma, skull surface, no angle): 0.80 mm posterior, 1.4 mm lateral, 2.5 mm ventral. The cannula was secured to the skull using dental acrylic and jeweler’s screws as anchors. Rats were allowed to recover for 5–7 days before food restriction procedures were implemented, after which cocaine self-administration training began the next day.

### Cocaine self-administration

Rats were trained to self-administer cocaine in behavioral chambers (Med-Associates Inc.) equipped with two levers, one active and one inactive. Pressing the active lever resulted in a cocaine infusion (0.31 mg/86 μL/5 s) on a fixed interval (FR1–20 s). A 20 s time out period was imposed to prevent overdose. A cue light (5 s) above the active lever and tone (4 kHz, 78 dB, 5 s) was paired with each cocaine infusion, and this light-tone cue was later used to trigger relapse on the cue-induced reinstatement test (see below). Pressing the inactive lever produced no consequence. Self-administration training occurred in daily 2 h sessions, 5 or 6 days a week, for a total of 14 sessions.

### Microinjection procedures

Microinfusions of SB203580 (5 nmol) or vehicle were administered through chronic indwelling cannulas aimed at the right ventricle. Microinjectors (33 gauge) were attached to Hamilton syringes via PE50 tubing and extended 1 mm beyond the tip of the cannula. Microinfusions were administered at a constant flow rate (5 µl over 5 min), and injectors remained in place an additional minute to allow diffusion. Rats were then immediately injected with U50 (5 mg/kg, i.p.) and returned to their home cage for 30 min before placement in behavioral chambers.

### Abstinence and extinction

Following self-administration, rats remained in the home cage for a 7-day forced abstinence period, in which no behavioral training or cocaine exposure occurred. Rats were weighed and handled every few days, and extinction training took place following the abstinence period. Extinction was conducted 5 days/week in 1 h sessions for a total of 10 sessions. During extinction, rats were returned to the same behavioral chamber as before, but cocaine and the light-tone cues were unavailable. Certain experimental groups received a total of 9 extinction sessions, since they either remained in their home cage on the U50 exposure day (U50-HC group), or were returned to the behavioral chambers after U50, but were not given access to levers (U50-NL group). Thus, their extinction day 1 began a day later than other groups. For graphical and statistical purposes, data were aligned to extinction day 1 for all groups.

### Cue-induced reinstatement testing

Following extinction, rats were placed in the behavior chambers for a 1 h cue-induced reinstatement test. A single presentation of the light-tone cue, previously paired with cocaine, was delivered at the beginning of the session serving to trigger reinstatement. Subsequently, the light-tone cue (but not cocaine) was contingent on active lever presses (FR1–20 s).

### Cocaine-primed reinstatement testing*

After the 1 h cue-induced reinstatement test, rats were allowed to continue responding for cocaine cues an additional 1 h, allowing for cue extinction. Then, rats were briefly removed from the behavioral chambers and administered an injection of cocaine (10 mg/kg, 1 ml/kg, i.p.) and returned to the behavioral chambers for an additional 1 h of testing. The cocaine-paired tone-light cues were available (FR1–20 s) throughout the entire session.

*Conducted only in Experiments 3 & 4

### Histology

Following completion of the experiment, rats with intracranial guide cannulas were transcardially perfused with 10% buffered formalin; brains were removed and sectioned at 40 μm increments. Sections were mounted onto slides and stained with neutral red. Placement of intracranial cannulas in the right lateral ventricle was verified for each rat by light microscopy.

### Statistical analyses

Lever pressing data were analyzed using two-way analyses of variance (ANOVAs) with appropriate post-hoc comparisons as specified in the Results. Experiments were conducted and analyzed separately, as reported in the Results; however, we also analyzed the pooled data from the first three experiments in a single 2-way ANOVA with Tukey’s post-hoc comparisons (Supplemental Table S1), which allows for certain additional comparisons to be made between groups across experiments. All statistical analyses were conducted with an alpha threshold of 0.05, and all data are reported as mean ± SEM.

## Results

Total cocaine intake (in mg/kg) at the end of cocaine self-administration was equivalent across all treatment groups before treatments were administered (Supplemental Figure S1). Furthermore, all groups showed equivalent extinction over the last 7 days of training and significant cue-induced reinstatement relative to the last extinction session (Supplemental Table S2). For Experiments 3 and 4, all groups showed significant within-session extinction over the 2 h prior to the cocaine priming injection and significant cocaine-primed reinstatement (Supplemental Table S3). Below we focus on differences between groups.

Experiment 1: Pre-treatment with U50 on extinction day 1 reduces cue-induced reinstatement of cocaine seeking 2 weeks later.

**Fig. 1:**
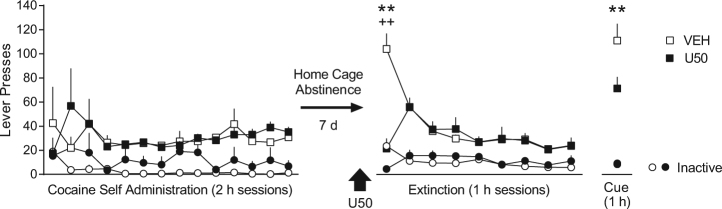
Pre-treatment with U50 on extinction day 1 reduces cue-induced reinstatement of cocaine seeking 2 weeks later. Following 2 weeks of cocaine self-administration and 7 days of home cage abstinence, rats (*n* = 7) were administered a single dose of U50 (5 mg/kg, i.p.) or VEH (*n* = 8) 30 min prior to the first extinction session. After extinction training was complete (10 sessions), rats underwent a cue-induced reinstatement test. U50 significantly reduced extinction responding on the first extinction session and 2 weeks later when challenged with cocaine cues (**p < 0.01 active lever, ^++^*p* < 0.01 inactive lever, comparing U50 to VEH)

Treatment groups (VEH or U50) were assigned in a pseudo-random fashion, balanced on response rates during acquisition of cocaine self-administration. Following cocaine self-administration, rats remained in their home cage for 7 days before extinction training commenced. 30 min prior to placement in the operant chambers for the first extinction training session, rats were administered vehicle (VEH) or U50 (5 mg/kg, i.p.). A two-way ANOVA over extinction/reinstatement identified a significant interaction between time and treatment for active lever responding [*F* (9, 117) = 9.46, *p* < 0.0001], and Sidak’s post-hoc comparisons indicated that U50 significantly reduced cocaine seeking on the first day of extinction (*p* < 0.0001; Fig. 1). U50 treatment also reduced inactive lever pressing on this day (Interaction: [*F* (9, 117) = 4.95, *p* < 0.0001], Sidak’s post-hoc, *p* < 0.0001; Fig. 1).

This acute reduction in cocaine seeking on extinction day 1 may be due to a combination of factors, including possible analgesia [9,10], dysphoria and/or aversion [7,20], and sedation or locomotor impairment [9,10], all of which have been reported with similar doses of U50. Notably, no differences between groups were found over the remainder of extinction training, but a marked reduction in cue-induced reinstatement, specific to the active lever, was observed in rats that received U50 2 weeks prior (*p* < 0.01; Fig. 1). We hypothesized that this reduction in cocaine seeking was due to an aversive counterconditioning process induced by U50-aversion on extinction day 1. This aversive memory may compete with the rewarding memory of the cocaine cues to reduce cocaine seeking. The next experiment was designed to test this theory.

Experiment 2: The long-term therapeutic effect of U50 on cue-induced reinstatement depends on extinction day 1 training and signaling through p38 MAPK.

If the long-term therapeutic effect of U50 treatment from Experiment 1 is mediated by an aversive counterconditioning process, the effect should depend on extinction training during U50 exposure. We also hypothesized that this aversive state would be mediated by p38 MAPK, which is required for U50-CPA [7,13]. For this experiment, we compared three treatment groups: (1) U50-HC rats that received U50 in the home cage instead of extinction day 1 training, (2) a group that received vehicle icv prior to U50 (VEH + U50) and extinction day 1 training, and (3) a group that received the p38 MAPK inhibitor SB203580 icv prior to U50 (SB + U50) and extinction day 1 training.

**Fig. 2:**
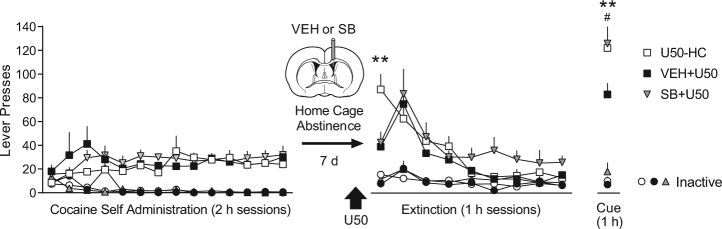
The long-term therapeutic effect of U50 on cue-induced reinstatement depends on extinction day 1 training and signaling through p38 MAPK. Following 2 weeks of cocaine self-administration and 7 days of home cage abstinence, rats were administered a single dose of U50 (5 mg/kg, i.p.) 30 min prior to the first extinction session, or in the home cage (U50-HC; *n* = 8). Extinction groups received vehicle (*n* = 6) or SB203580 (*n* = 7) microinfusions (icv) just prior to U50. U50 significantly reduced extinction responding on the first extinction session in both groups (**p < 0.01 active lever, compared to U50-HC), regardless of p38 MAPK inhibition. There were no differences over subsequent extinction sessions. Rats underwent a cue-induced reinstatement test (2 weeks post-treatment), and prior treatment with SB + U50 on the first extinction session prevented U50’s long-lasting therapeutic reduction in cocaine seeking (**p < 0.01 active lever, comparing VEH + U50 to SB + U50; #*p* < 0.05 active lever, comparing U50-HC to VEH + U50)

All experimental parameters were the same as Experiment 1, until the day of U50 treatment. On that day, U50-HC rats remained in their home cage after U50 treatment. VEH + U50 and SB + U50 groups were returned to behavioral chambers for extinction day 1 training 30 min after U50 injection. VEH or SB203580 was infused icv immediately prior to U50. A two-way ANOVA over extinction/reinstatement identified a significant interaction between time and treatment for active lever responding [*F* (18,162) = 2.27, *p* < 0.01], and Tukey’s post-hoc comparisons indicated that U50 significantly reduced cocaine seeking on the first day of extinction (*p* < 0.01 compared to U50-HC; Fig. 2), regardless of p38 MAPK inhibition. As before, no differences between groups were found over the remainder of extinction training. However, a significant reduction in cue-induced reinstatement was observed in the VEH + U50 group relative to the SB + U50 group (*p* < 0.01; Fig. 2) and the U50-HC group (*p* < 0.05; Fig. 2), indicating that both extinction training and p38 MAPK signaling at the time of U50 exposure are required for the long-term therapeutic effect of U50 on reinstatement. All effects were specific to the active lever.

Experiment 3: The long-term therapeutic effect of U50 on cue-induced reinstatement depends on operant, not contextual, extinction on extinction day 1.

We hypothesized that the long-term therapeutic effects of U50 on reinstatement of cocaine seeking would depend on either contextual or operant extinction on extinction day 1. To test this, we treated two groups of rats with U50 (as in previous experiments) prior to the first extinction session where levers were available as usual (U50 group), or withheld (i.e., no levers; U50-NL group). If the long-term therapeutic effect of U50 depends on contextual extinction, no differences between groups would be expected; if the U50 effect depends on operant extinction, the U50-NL group would be expected to reinstate more than the U50 group.

**Fig. 3:**
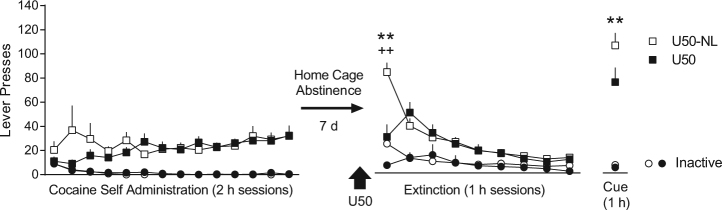
The long-term therapeutic effect of U50 on cue-induced reinstatement depends on operant, not contextual, extinction on extinction day 1. Using the same experimental design as previous experiments, a single dose of U50 (5 mg/kg, i.p.) was administered 30 min prior to the first extinction session. The U50 group (*n* = 7) had access to levers as usual, but the U50 No Lever (U50-NL; *n* = 7) group did not. U50-NL significantly differed from U50 on the first extinction session with access to levers. Prior treatment with U50, only when levers were present, resulted in a long-lasting therapeutic reduction in cocaine seeking on the cue-induced reinstatement test (**p < 0.01 active lever, ^++^*p* < 0.01 inactive lever, comparing U50 to U50-NL)

Rats were administered U50 (5 mg/kg, i.p.) 30 min prior to extinction day 1 as usual (U50 group), or with no levers extended (U50-NL group) to allow contextual extinction, but not operant extinction to occur. A two-way ANOVA over extinction/reinstatement identified a significant interaction between time and treatment for active lever responding [*F* (9, 108) = 6.62, *p* < 0.0001], and Sidak’s post-hoc comparisons indicated that U50 significantly reduced cocaine seeking on the first day of operant extinction (*p* < 0.0001 compared to U50; Fig. 3), with no differences over subsequent extinction days. As in Experiment 1, this effect was also seen on the inactive lever (Interaction: [*F* (9, 108) = 2.71, *p* < 0.01], Sidak’s post-hoc, *p* < 0.01; Fig. 3), and no differences between groups were observed over the remainder of extinction training. However, a significant reduction in cue-induced reinstatement, specific to the active lever, was observed in the U50 group relative to the U50-NL group (*p* < 0.01; Fig. 3), indicating that operant, not contextual, extinction is required for U50’s long-term reduction in reinstatement.

We also tested whether the long-term therapeutic effect of U50 would extend from reinstatement triggered by conditioned stimuli (the light-tone cocaine-cue) to the unconditioned stimulus (cocaine). Thus, immediately following the 1 h cue-induced reinstatement test, rats were allowed to continue responding for an additional hour in order to extinguish responding to the cue (Supplementary Figure S2A). Rats were then briefly removed from chambers, administered a cocaine priming injection (10 mg/kg), and returned for a final 1 h cocaine-induced reinstatement test (cues available throughout). A two-way ANOVA analyzing cumulative active lever presses over these 2 h confirmed a significant interaction between time and treatment [*F* (23,276) = 1.77, *p* < 0.05], and Fisher’s LSD post-hoc tests indicated the U50 group reinstated significantly less than the U50-NL group during the last 35 min of the cocaine-primed reinstatement test (*p* < 0.05, Supplementary Figure S2A), and this difference was specific to the active lever.

Experiment 4: Lithium chloride acutely reduces cocaine seeking on extinction day 1, but does not have a long-term therapeutic effect on cue-induced reinstatement.

**Fig. 4:**
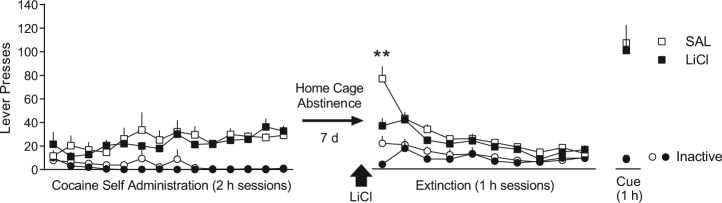
Lithium chloride acutely reduces cocaine seeking on extinction day 1, but does not result in a long-term therapeutic effect on cue-induced reinstatement. This experiment examined whether the therapeutic reductions in cocaine seeking observed after U50-extinction treatment could be mimicked by another aversive compound, lithium chloride (LiCl). Thus, the experimental design matched that of Experiment 1, except instead of U50, rats (*n* = 7) were injected with LiCl (127 mg/kg) or saline (*n* = 8) 30 min prior to the first extinction session. Similar to U50, LiCl reduced cocaine seeking on extinction day 1 relative to saline (SAL) controls, but had no long-lasting effects on subsequent extinction or cue-induced reinstatement. (**p < 0.01 active lever, compared to SAL)

We hypothesized that the long-term therapeutic effects of U50 on cocaine seeking would be a unique feature of counterconditioning with KOR agonists, compared to other aversive compounds. LiCl is often used to countercondition or devalue rewards through its known ability to induce gastric malaise [32,33]. This study was set up to resemble Experiment 1, except that rats received LiCl (127 mg/kg, i.p.) instead of U50. A two-way ANOVA over extinction/reinstatement identified a significant interaction between time and treatment for active lever responding [*F* (10,130) = 2.01, *p* < 0.05], and Sidak’s post-hoc comparisons indicated that LiCl acutely reduced cocaine seeking on extinction day 1 (*p* < 0.001, Supplementary Figure S2B), similar to U50. However, unlike U50, LiCl resulted in no long-term therapeutic effects on cocaine seeking, either on cue-induced reinstatement (Fig. 4) or cocaine-primed reinstatement (Supplementary Figure S2B). No differences were observed for inactive lever responding. These data demonstrate that U50 has unique pharmacological properties that elicit long-term reductions in relapse, and this effect is not mimicked by LiCl.

## Discussion

The present study identifies the therapeutic efficacy of activating KORs in conjunction with extinction training to reduce cocaine relapse induced by cocaine-conditioned cues. This effect requires p38 MAPK signaling in brain, and thus likely results from U50-aversion [7]. Because the effect requires concomitant extinction training, involving both extinction of the contextual cues of the cocaine-paired behavioral chamber and operant extinction, an aversive counterconditioning process can account for these observations. That the long-term therapeutic effect of U50 was not mimicked by another aversive compound, namely LiCl, suggests KOR agonists have unique properties for reducing long-term relapse rates after exposure therapy. The ability of a single treatment to elicit such long-term therapeutic effects is rarely reported, but is consistent with an aversive counterconditioning hypothesis. Our results demonstrate the ability of KOR agonists to act as punishers of cocaine-seeking behavior [25], and call for a re-examination of KOR agonists and their dysphoric “side effects” as potential therapeutic tools in translational addiction therapy.

### KORs, dysphoria, and p38 MAPK

Both stress and cocaine exposure elicit changes in behavior by releasing dynorphin and activating KORs, thus sharing a common substrate in central KOR systems [19,20]. A single, acute stressor can produce long-lasting increases in KOR signaling within the VTA, which promotes drug seeking [23,35]. By contrast, repeated cocaine is required to elicit the aversive states characteristic of cocaine withdrawal, and KOR antagonists can prevent these symptoms [2,36,37]. Consistent with this, KOR antagonists reduce stress-induced, but not cocaine-primed, reinstatement [3]. On the other hand, KOR agonists have shown promise in reducing cocaine self-administration and cocaine-primed reinstatement [4,5,24], suggesting a fundamental difference in the therapeutic efficacy of KOR agents depending on whether cocaine is systemically present.

There is a long history demonstrating the therapeutic potential of KOR agonists to reduce drug seeking, but most of these studies have focused on counteracting the effects of acute drug exposure, for example through the known ability of KOR agonists to reduce dopamine release [38–40]. To our knowledge, there are no reports examining the therapeutic potential of KOR agonists as extinction therapy adjuncts. The dose of U50 we used for these experiments has been reported to induce CPA in both mice and rats, an effect which depends on p38 MAPK activation [7,29]. However, this dose may also produce analgesic and sedative effects, which become more pronounced with higher doses [9]. Indeed, the acute reduction in cocaine seeking observed on extinction day 1, during U50 exposure, was likely not mediated by p38 MAPK, although we cannot exclude the possibility that higher doses of the p38 MAPK inhibitor may have been effective. However, this same dose of the p38 MAPK inhibitor effectively prevented the long-term reduction in relapse after U50 treatment. Thus, the acute effects of U50 reported here were likely mediated by other signaling cascades triggered by KOR agonism, such as pERK or JNK [19].

### Long-term therapeutic effect of U50-counterconditioning on reinstatement of cocaine seeking

The long-lasting therapeutic effect of U50 on reinstatement of cocaine seeking is most parsimoniously explained by U50-counterconditioning. That U50 can induce CPA [7,29] indicates its ability to induce an aversive memory, which may effectively counteract the rewarding memory associated with the conditioned cocaine-seeking response. Interestingly, U50 counterconditioning specifically required operant, but not contextual, extinction. Because the operant response is required for both cue-induced and cocaine-primed reinstatement, this could explain why both forms of reinstatement were reduced in U50 rats (Supplemental Figure S2). Although U50 treatment on extinction day 1 significantly reduced reinstatement compared to controls, U50 rats still exhibited significant reinstatement relative to extinction baseline, indicating that the memory for the cocaine-cues was still intact and capable of driving the conditioned seeking response. Siegmund and Wotjak [41] have suggested that there are two main components driving a conditioned response—a categorical component reflecting the associative memory, and a sensitized component that effectively amplifies this response. We suggest that U50 may act specifically on the sensitized component, perhaps by interfering with the frustrative non-reward known to be characteristic of the first extinction experience [42].

These long-term therapeutic effects of U50 exhibit some similarity to lithium chloride (LiCl)-induced devaluation of rewards. LiCl induces gastric malaise, which when paired with a normally rewarding substance like sucrose or cocaine, reduces motivation to consume and/or seek the substance thereafter [43]. Although LiCl treatment acutely reduced cocaine seeking on extinction day 1, it failed to elicit the long-term reduction in relapse seen with U50. Thus, while both compounds induce aversive interoceptive states acutely, U50 may be unique in its ability to elicit long-term therapeutic effects from aversive counterconditioning in this manner, perhaps owing to its selective association with the operant response. Whereas both LiCl and U50 are capable of inducing CPA [7,44], LiCl produces long-lasting effects on reinstatement only when administered during reconsolidation of a cocaine conditioned place preference memory [44]. It is worth noting that the immediate early gene and transcription factor Zif268, which has been associated with memory reconsolidation [45], is downstream of KOR signaling through p38 MAPK [7,14]. Thus, it is possible the long-term therapeutic effects of U50 may involve a memory updating process that links U50-aversion with the operant response, possibly through a reconsolidation-like mechanism (i.e., Zif268). Notably, however, our behavioral protocol induces extinction, which generally opposes reconsolidation. Our results demonstrate that U50, but not LiCl, administered in conjunction with extinction training produces long-lasting therapeutic reductions in relapse.

KOR agonists have long been known to elicit therapeutic reductions in drug taking and/or seeking for numerous drugs of abuse, ranging from psychostimulants to opiates [25]. Most of these studies report acute effects on drug self-administration and/or stress-induced reinstatement of drug seeking before washout of the KOR agonist. Here we report therapeutic effects of a single injection of a KOR agonist when paired with extinction training. Our data suggest this treatment works through aversive counterconditioning of the drug-seeking response, as p38 MAPK signaling is required at the time of U50 exposure, and is also a requirement for U50-CPA [7,13]. Our data call for a re-examination of full KOR agonists, or KOR agonists biased toward the beta arrestin-p38 MAPK pathway [15], as viable therapeutics for counterconditioning cocaine cues and reducing rates of relapse.

## Electronic supplementary material

Supplemental Material
