# Supplementary material for: A single, extinction-based treatment with a kappa opioid receptor agonist elicits a long-term reduction in cocaine relapse
Source: Neuropsychopharmacology. 2018 Feb 22;43(7):1492–7. doi: 10.1038/s41386-017-0006-4 (PMC5983548; doi:10.1038/s41386-017-0006-4)
Supplement: Supplementary file 1 — Supplemental Material [file 41386_2017_6_MOESM1_ESM.pdf]

Supplementary Table S1

	TESTS	
**TREATMENT**	**Ext Day 1**	**Cue Test**
VEH vs U50	****	**
VEH vs U50 HC	ns	ns
VEH vs U50 NL	ns	ns
VEH vs VEH+U50	****	ns
VEH vs SB+U50	****	ns
U50 vs U50 HC	****	****
U50 vs U50 NL	****	*
U50 vs VEH+U50	ns	ns
U50 vs SB+U50	ns	****
U50 HC vs U50 NL	ns	ns
U50 HC vs VEH+U50	***	*
U50 HC vs SB+U50	**	ns
U50 NL vs VEH+U50	**	ns
U50 NL vs SB+U50	**	ns
VEH+U50 vs SB+U50	ns	**

Table S1. Tukey's post-hoc significance levels from a two-way ANOVA of combined Experiments 1, 2 and 3 data. U50 treatment groups from Experiments 1 and 3 were not different and thus pooled into a single U50 group. A two-way ANOVA over active lever responding across all treatment groups revealed a significant interaction [$F(45, 396) = 4.31$, $p < 0.0001$] with Tukey's post hoc comparisons as indicated. **** $p < 0.0001$, *** $p < 0.001$, ** $p < 0.01$, and * $p < 0.05$. Comparisons reported in the main text are highlighted.

Supplementary Table S2

	TREATMENT	FIRST DRUG-FREE EXTINCTION DAY	LAST EXTINCTION DAY	CUE-INDUCED REINSTATEMENT
**EXPERIMENT 1**				
Active Lever	VEH	56.0 ± 5.7 **	24.1 ± 6.5	111.0 ± 14.0 **
$F_{(9,117)} = 9.46$, $p < 0.0001$	U50	55.7 ± 7.9 **	23.7 ± 5.9	71.3 ± 9.7 **
Inactive Lever	VEH	11.3 ± 2.0	5.9 ± 1.5	8.5 ± 1.7
$F_{(9,117)} = 4.95$, $p < 0.0001$	U50	4.6 ± 1.2	11.1 ± 4.6	9.3 ± 1.8
**EXPERIMENT 2**				
Active Lever	U50 HC	62.6 ± 9.8 **	12.8 ± 1.9	122 ± 23.4 **
$F_{(18,162)} = 2.27$, $p < 0.01$	VEH+U50	75.0 ± 7.4 **	15.2 ± 3.1	83.0 ± 9.4 **
	SB+U50	83.4 ± 21.0 **	25.85 ± 5.6	125.9 ± 17.4 **
Inactive Lever	U50 HC	12.3 ± 2.7	6.5 ± 1.6	10.3 ± 3.5
ns	VEH+U50	20.7 ± 4.5	6.7 ± 1.2	7.2 ± 2.4
	SB+U50	20.1 ± 6.9	8.9 ± 3.5	18.1 ± 7.6
**EXPERIMENT 3**				
Active	U50 NL	39.4 ± 6.1 **	12.9 ± 1.9	106.6 ± 10.3 **
$F_{(9,108)} = 6.616$, $p < 0.0001$	U50	50.4 ± 8.7 **	11.4 ± 1.1	75.9 ± 12.2 **
Inactive	U50 NL	12.3 ± 3.3	6.3 ± 1.6	6.7 ± 2.0
$F_{(9,108)} = 2.717$, $p < 0.01$	U50	12.9 ± 4.3	1.4 ± 0.4	4.6 ± 1.5
**EXPERIMENT 4**				
Active	SAL	42.7 ± 4.8 **	13.1 ± 3.0	107.0 ± 15.3 **
$F_{(10,130)} = 2.014$, $p < 0.05$	LiCl	41.9 ± 6.6 **	16.4 ± 3.8	100.6 ± 16.6 **
Inactive	SAL	20.6 ± 5.2	9.6 ± 3.3	8.8 ± 1.9
ns	LiCl	17.4 ± 5.5	9.0 ± 5.9	8.1 ± 2.6

Table S2. Extinction and cue-induced reinstatement lever pressing for all experiments. Two-way ANOVAs for each experiment revealed significant interactions for active and inactive levers as indicated. Lever pressing data are expressed as mean ± SEM. ** p < 0.01 Sidak's post-hoc compared to the last extinction day.

Supplementary Table S3

	TREATMENT	FIRST 15 MIN CUE	LAST 15 MIN CUE REINSTATEMENT	FIRST 15 MIN COCAINE TEST
**EXPERIMENT 3**				
Active	U50 NL	45.3 ± 7.2 **	10.1 ± 1.4	65.4 ± 17.3 **
$F_{(11,132)} = 10.97$, $p < 0.0001$	U50	33.1 ± 7.0 *	6.1 ± 1.9	45.1 ± 12.4 **
Inactive	U50 NL	4.0 ± 1.2	0.3 ± 0.3	2.7 ± 1.5
ns	U50	3.0 ± 0.8	0.1 ± 0.1	0.8 ± 0.4
**EXPERIMENT 4**				
Active	SAL	49.6 ± 6.3 **	7.5 ± 2.1	58.0 ± 16.6 *
$F_{(11,143)} = 10.04$, $p < 0.0001$	LiCl	45.0 ± 10.2 *	8.0 ± 1.7	68.7 ± 19.0 **
Inactive	SAL	6.5 ± 1.5 **	0.6 ± 0.3	0.9 ± 0.4
$F_{(11,143)} = 3.540$, $p < 0.001$	LiCl	2.9 ± 0.5	0.5 ± 0.3	0.6 ± 0.3

Table S3. Cue extinction and cocaine-primed reinstatement lever pressing for Experiments 3 and 4. Two-way ANOVAs of active versus inactive lever presses were conducted over the time course (15-min binned data) of the 3 h reinstatement session in Experiments 3 and 4, separately. Significant effects of time were observed as indicated. Lever press data are expressed as mean ± SEM. * p<0.05, **p<0.01 Sidak's post-hoc compared to the last bin before the cocaine prime.

Supplementary Figure S1

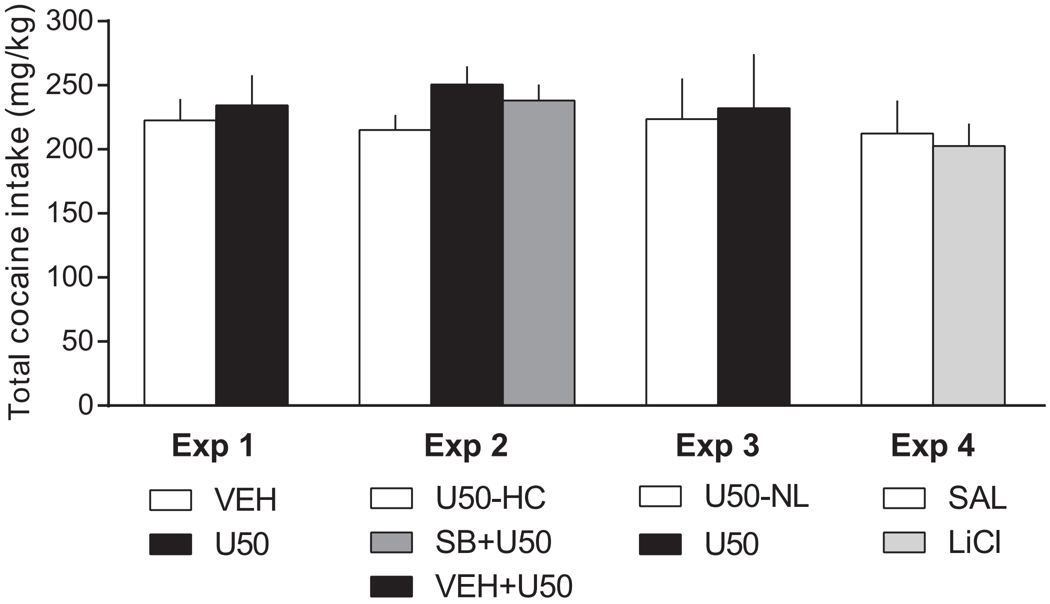

Figure S1. Total cocaine intake across treatment groups. Total cocaine intake was calculated in mg/kg for each rat on a daily basis, summated across all 14 cocaine self-administration sessions, and graphed as mean ± SEM. A one-way ANOVA revealed no differences between groups.

Supplementary Figure S2

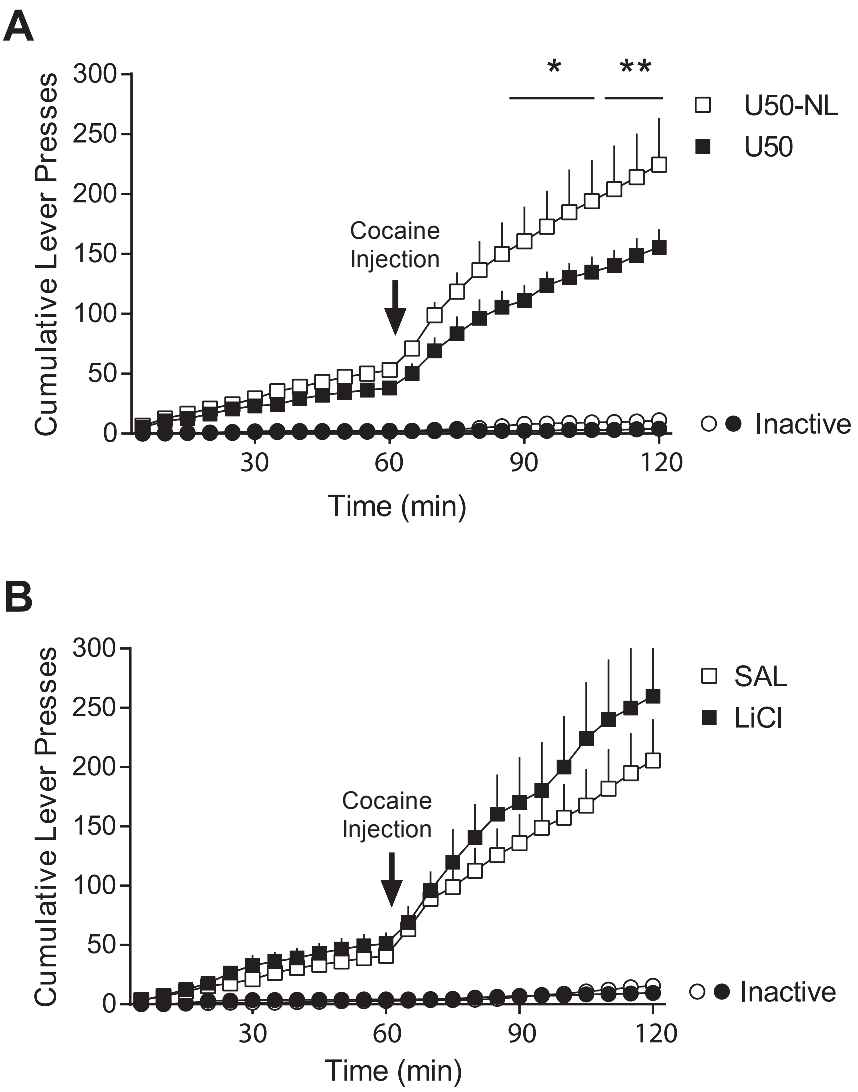

Figure S2. Pre-treatment with U50 on extinction day 1 reduces cocaine-primed reinstatement of cocaine seeking 2 weeks later. Animals in Experiment 3 (A) and Experiment 4 (B) underwent a prolonged cue-induced reinstatement test, including an additional hour to extinguish responding for the cues, followed by a cocaine priming injection (10 mg/kg, i.p.) and an additional hour of testing (cues available throughout). The time course of responding over these 2 additional hours of the test are shown as cumulative lever presses over time, and all groups exhibited significant cocaine-primed reinstatement (see text and Table S3 for statistics). A. A two-way ANOVA revealed a significant interaction [$F(23,276) = 1.77$, $p<0.05$] and group differences emerged during the last 35min of the test (* $p<0.05$; ** $p < 0.01$ Fisher's LSD post-hoc comparing U50 to U50-NL). No effects were found on cumulative inactive lever presses. B. No differences were observed between LiCl and SAL on either active or inactive lever pressing.
